# Study on the Tensile Properties and Influencing Factors of Superelastic SMAF-Reinforced PP/PVA-ECC Materials

**DOI:** 10.3390/ma19020263

**Published:** 2026-01-08

**Authors:** Yan Cao, Xiaolong Qi, Zhao Yang

**Affiliations:** 1 College of Creative Design, Wuchang University of Technology, Wuhan 430223, China; andycaoyan@163.com; 2 School of Urban Construction, Wuhan University of Science and Technology, Wuhan 430065, China; wy156182@163.com

**Keywords:** SMAF-ECC, Tensile property, PP fibers, PVA fibers, SMAF

## Abstract

To develop a cost-effective shape memory alloy fiber-reinforced engineered cementitious composite (SMAF-ECC) with excellent mechanical properties, polypropylene (PP) fibers were used to partially replace polyvinyl alcohol (PVA) fibers to prepare the ECC matrix, and superelastic shape memory alloy fibers (SMAFs) were incorporated to fabricate a novel SMAF-ECC. Uniaxial tensile tests were systematically performed to characterize the tensile mechanical properties of the composites, focusing on the effects of SMAF volume content and diameter. The results indicate that the optimal base ECC mix proportion is 0.8 vol.% PP fibers and 1.2 vol.% PVA fibers, achieving an ultimate tensile strain of 4.88% (only a 4.69% reduction compared to pure PVA-ECC) while significantly reducing material cost without sacrificing superior ductility. SMAF volume content and diameter notably influence the tensile performance of SMAF-ECC, with the specimen containing 0.2 mm diameter SMAFs at 0.2 vol.% exhibiting the best performance: initial cracking stress, ultimate tensile stress, and ultimate tensile strain are enhanced by 16.79%, 20.85%, and 2.87%, respectively, compared to pure ECC. This study provides a theoretical basis and parametric guidance for the engineering popularization and application of cost-effective SMAF-ECCs.

## 1. Introduction

Engineered cementitious composites (ECCs) are a type of fiber-reinforced ultra-high-toughness cementitious composite that exhibit high ductility, tensile strain hardening, and multiple-cracking behavior [1,2]. These excellent mechanical properties endow ECC with broad application prospects in engineering fields such as construction, transportation, and tunnels [3,4,5,6,7]. However, the currently widely used polyethylene (PE) fibers [8,9] and polyvinyl alcohol (PVA) fibers [10,11] are expensive, leading to increased initial material costs of ECC and limiting its popularization and application in practical engineering [12]. Therefore, it is crucial to develop cost-effective ECC materials [13,14]. Domestic polypropylene (PP) fibers have mature preparation technology and a low cost, only 1/16th of that of PVA fibers. Relevant studies have shown that polypropylene fiber-reinforced engineered cementitious composites (PP-ECCs) exhibit high tensile strength under uniaxial tension, with a strain reaching 3~5% [15]. However, as the bond strength and tensile strength of PP fibers in ECC are lower than those of PVA fibers, the tensile strain and toughness of PP-ECCs cannot meet the demands of wider engineering applications [16]. Thus, the hybrid reinforcement of ECC with PP and PVA fibers (PP/PVA-ECC) has attracted the attention of scholars. Pakravan et al. [17] replaced 20% (by volume) of ECC’s original fiber component with triangular PP fibers or low-modulus PVA fibers. This modification significantly enhanced the material’s strain capacity by 33% and 148%, respectively. Additionally, it ensured the material retained the strain hardening effect and multiple-cracking characteristics inherent to cementitious composites. Lin et al. [18] also found that when the volume content of PVA fibers was fixed at 1.0%, adding PP fibers with a volume content of 0.5%, 1.0%, and 1.5%, respectively, could increase the tensile strength of PP-PVA-ECC by 9.46%, 21.1%, and 57.7%, and the ultimate tensile strain by 22.1%, 59.3%, and 69.0%, respectively. However, while previous studies have demonstrated that the hybridization of PP and PVA fibers can enhance the mechanical properties of ECC, the material’s excellent toughness and high energy absorption capacity are typically achieved at the cost of extensive cracking and residual deformation. Such characteristics hinder the rapid repair and functional recovery of structures after loading [19].

Shape memory alloy (SMA) is a novel class of functional material exhibiting a unique shape memory effect or superelasticity, along with excellent fatigue resistance [20,21,22]. In particular, the flag-shaped hysteretic energy dissipation behavior and deformation recovery capability of superelastic SMA make it highly suitable for seismic energy dissipation and structural deformation recovery [23]. Several scholars have combined superelastic SMA bars with ECC to develop SMA-reinforced ECC (SMA-ECC), a composite with high ductility and self-healing properties [24,25]. Nevertheless, SMA tendons suffer from drawbacks including high cost, complex construction processes, and numerous internal defects, which restrict the widespread popularization and application of SMA-ECC [26,27].

Superelastic SMA fiber (SMAF) not only retains the excellent properties of SMA bars but also features a simpler construction process and more cost-effective price. In addition, SMAF exhibits uniform and random distribution within the matrix, making it more compatible with ECC matrix materials prone to large-scale cracking [28]. Therefore, the development of SMAF-reinforced ECC (SMAF-ECC) has garnered significant attention from researchers. Yang et al. [29] investigated the tensile mechanical properties of SMAF/PVA hybrid fiber-reinforced cementitious composites (SMAF/PVA-ECCs). The results indicate that the incorporation of SMAF significantly enhances the tensile properties of SMAF/PVA-ECC, with the ultimate tensile stress reaching 5.24 MPa. Chen et al. [30] examined the influence of SMAF content on the self-healing performance of SMAF-ECC via four-point bending tests. The findings reveal that as the SMAF content increases from 0.3% (volume content) to 0.7%, the crack closure efficiency of SMAF-ECC for cracks with a width less than 50 μm is significantly enhanced. Yang et al. [31] explored the closed crack performance of SMAF-ECC through uniaxial cyclic tensile tests. The results demonstrate that the maximum strain recovery rate and crack recovery rate of the test specimens reach 69% and 77%, respectively. Ali et al. [32,33] evaluated the mechanical properties of ECC materials blended with SMAF and PVA fibers and found that compared with ECC containing only PVA fibers, the tensile and flexural properties of the composites are significantly improved following the incorporation of SMAF.

Although the research findings on SMAF-ECC are promising, the high material cost restricts its practical engineering application. As revealed in the preceding sections, PP fibers can improve the tensile strength and tensile strain capacity of cementitious composites to a certain extent [15]. Although their reinforcing effect is inferior to that of PVA fibers, PP fibers exhibit remarkable cost advantages. To develop cost-effective SMAF-ECC materials with excellent mechanical properties, this study attempts to prepare PP/PVA-ECC by hybridizing low-cost PP and PVA fibers, followed by combination with SMAF to fabricate SMAF-ECC and realize its cost advantage. To investigate whether SMAF-ECC still retains favorable mechanical properties, this paper conducts research on the tensile performance of the material. Through uniaxial tensile tests, the tensile mechanical property indicators of SMAF-ECC are studied, and main influencing factors such as SMAF content and diameter are compared. The research results provide a theoretical basis for developing cost-effective SMAF-ECC materials with excellent mechanical properties and promoting their engineering application.

## 2. Mechanical Properties of Materials

### 2.1. Tensile Properties of SMAF

SMAFs were supplied by Jiangyin Renchang Nickel-Titanium New Material Co., Ltd. (Wuxi, China), with diameters of 0.2, 0.5, and 0.8 mm. The main chemical composition of the SMAF was 55.9% (mass fraction) Ni and 44.05% Ti. The tensile properties of SMAF are presented in Table 1, which were provided by the manufacturer.

### 2.2. Tensile Properties of PP/PVA-ECC

#### 2.2.1. Raw Materials

PP/PVA-ECC (hereinafter referred to as ECC) was fabricated using the following raw materials: Panlongshan P·I 52.5 Portland cement, as its chemical composition meets the GB 175-2023 standard [34]; grade I fly ash (main components are SiO_2_, alumina, Fe_2_O_3_, etc.; density: 2.55 g/cm^3^, fineness: 16 μm, water absorption is 89%); gray crystalline quartz sand (main component is SiO_2_; silicon content 98.6%, density 1.65 g/cm^3^, particle size 70–110 mesh); polycarboxylate water reducer (water content 2.2%). PP fibers were supplied by China Textile Fibers Jiankaitai Technology Co., Ltd. (Xiamen, China), and PVA fibers were provided by Kuraray Co., Ltd., Tokyo, Japan. The mechanical properties of the PP and PVA fibers are presented in Table 2, provided by the manufacturer.

#### 2.2.2. Specimen Design and Production

**Figure 1:**
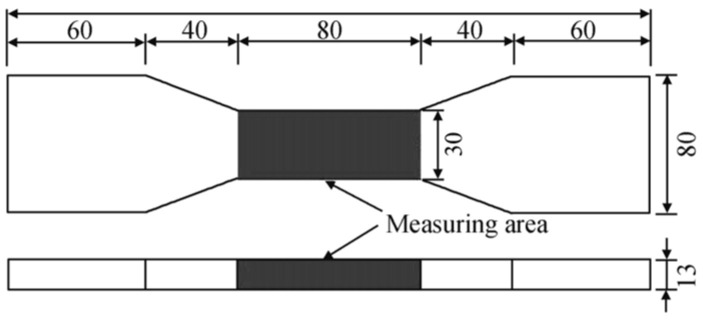
Schematic diagram of the dimensions of tensile specimen (size: mm).

In accordance with the “Standard test method for the mechanical properties of ductile fiber reinforced cementitious composites” (JC/T2461-2018) [35], the tensile specimens were designed as dog-bone-shaped, and the specific dimensions of the specimens are illustrated in Figure 1. Among them, the size of the middle test section of the dog-bone-shaped specimen is as follows: the gauge length is 80 mm, the section width is 30 mm, and the thickness is 13 mm.

For the ECC tensile performance test, referring to Zhu et al. [36]’s research on PP fiber-reinforced cement-based composites and integrating the previous research findings of the research group [29,31], four mix proportion schemes were designed, including ECC-0 (without PP fibers), as presented in Table 3. Three replicate specimens were prepared for each mix proportion.

During specimen preparation, cement, fly ash, quartz sand, and water reducer were first weighed accurately and added to a JJ-5 cement mortar mixer. The powder materials were homogenized via dry mixing for 2 min. Subsequently, 50% of the total mixing water was added, and the mixture was stirred at low speed for 1 min; the remaining water was then added, followed by another 1 min of stirring at low speed, resulting in a mixture with good fluidity. With the mixer still operating at low speed, PVA fibers and PP fibers were incorporated in batches, and the mixing speed was switched to high for 3 min to ensure uniform fiber dispersion. After thorough mixing, the fresh mixture was poured into dog-bone-shaped molds and vibrated for compaction and shaping. After 24 h of initial curing, the specimens were demolded. Finally, all demolded specimens were transferred to a standard curing room for 28 days of curing under standard conditions.

#### 2.2.3. Loading Equipment and Measurement Method

**Figure 2:**
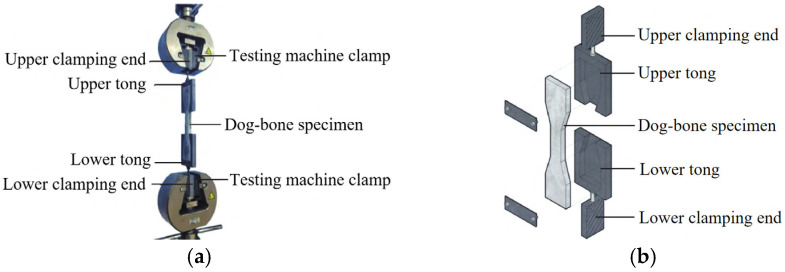
Schematic diagram of uniaxial tensile test setup and tensile fixture: (**a**) uniaxial tensile test setup; (**b**) tensile fixture.

Uniaxial tensile tests were conducted using a WD-PD6305 universal testing machine (Jinan puye Electromechanical Technology Co., Ltd., Jinan, China). The tests adopted a displacement-controlled loading mode with a preset loading rate of 0.5 mm/min. Load and displacement data were automatically recorded and stored by the testing machine, and stress and strain were calculated based on the actual dimensions of the specimens. To avoid abnormal fracture and slippage of specimens caused by uneven stress distribution, a specialized tensile fixture device was designed and fabricated. Schematic diagrams of the test setup and the tensile fixture are illustrated in Figure 2.

#### 2.2.4. Test Results and Analyses

**Figure 3:**
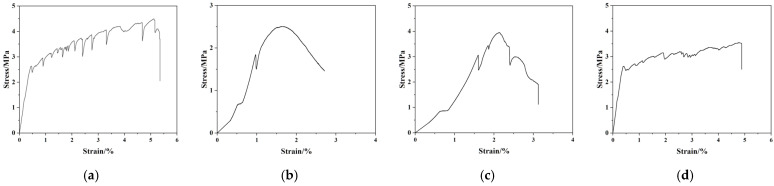
Tensile stress–strain curves of ECC specimens with different mix ratios: (**a**) ECC-0; (**b**) ECC-1; (**c**) ECC-2; (**d**) ECC-3.

The stress–strain curves and characteristic parameters of ECC tensile specimens with different mix ratios are presented in Figure 3 and Table 4. As observed in Figure 3, the tensile stress–strain curves of ECC-1 and ECC-2 specimens exhibit a two-stage development pattern. In contrast, ECC-3 specimens and the control group (ECC-0) show a three-stage development trend and exhibit distinct strain hardening behavior. Comparing Figure 3a–d, it can be found that as the PP fiber content decreases and the PVA fiber content increases, the curves exhibit more frequent fluctuations, indicating an increase in the number of microcracks. This is attributed to the higher tensile strength of PVA fibers compared to PP fibers: after PP fibers fracture, PVA fibers can still transfer part of the load to the uncracked matrix, leading to the formation of more and finer microcracks. As shown in Table 4, among all tested groups, ECC-1 specimens exhibit the lowest ultimate tensile strength and ultimate tensile strain, reaching 2.51 MPa and 2.14%, respectively, corresponding to the poorest tensile performance. For ECC-2 specimens, the ultimate tensile strain is only 2.39%, which is significantly lower than the 5.12% of the control group (ECC-0), indicating inferior tensile properties. Notably, ECC-3 specimens demonstrate a higher ultimate tensile strength (3.55 MPa) and ultimate tensile strain (4.88%). Compared with ECC-0 specimens, the ultimate tensile strain of ECC-3 is only reduced by 4.69%, retaining excellent ductility. The above results indicate that the third mix proportion scheme—with PP and PVA fiber volume contents of 0.8% and 1.2%, respectively—yields the optimal tensile performance for ECC. This mix proportion not only maintains the excellent tensile strain capacity of cement-based composites but also reduces the material cost effectively.

## 3. Tensile Performance Test of SMAF-ECC

### 3.1. Experimental Design

#### 3.1.1. Specimen Design and Production

**Figure 4:**
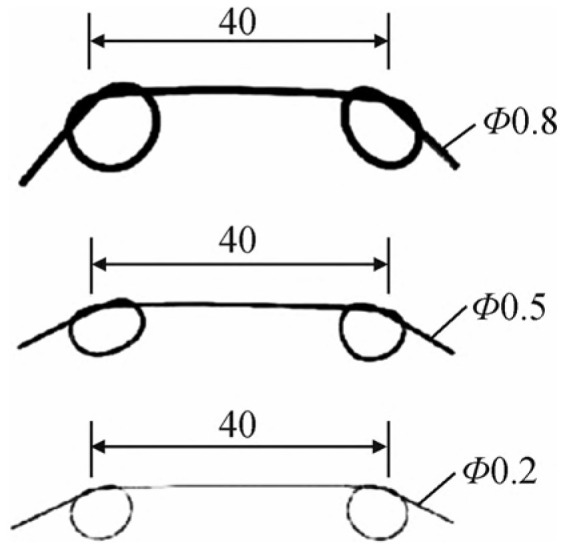
SMAF with knotted ends (size: mm).

SMAF-ECC specimens adopted the same dimensions as the ECC tensile specimens, as illustrated in Figure 1. They were fabricated based on the optimal mix proportion identified from the ECC tensile tests—with PP and PVA fiber volume contents of 0.8% and 1.2%, respectively. According to the previous research findings of the research group [29], the optimal length of SMAF is approximately 40 mm, and SMAF-ECC exhibits the best performance when the fiber ends are designed as knotted configurations. Therefore, the SMAF used in this study had a fixed length of 40 mm and knotted ends, as shown in Figure 4. Uniaxial tensile tests were conducted to investigate the effects of SMAF content and diameter on the tensile properties of SMAF-ECC. The test groups consisted of three SMAF volume contents (0.2%, 0.3%, and 0.4%) and three SMAF diameters (0.2, 0.5, and 0.8 mm). The control groups included ECC specimens without SMAF and specimen 0-S-0.2-0.2 (PVA-ECC matrix, SMAF diameter: 0.2 mm, SMAF volume content: 0.2%). The detailed specimen designations and parameters are presented in Table 5.

The preparation procedure of the ECC mixture refers to Section 2.2.2 of this paper. After obtaining the ECC mixture, SMAF was added in two batches and stirred thoroughly to ensure uniform fiber dispersion. The fresh mixture was then poured into dog-bone-shaped molds, vibrated for compaction and shaping, and demolded after 24 h of initial curing. Finally, the specimens were transferred to a standard curing chamber for 28 days of curing under standard conditions.

#### 3.1.2. Loading Equipment and Measurement Method

For the loading equipment of this test, the WD-PD6305 universal testing machine was still employed. The loading system and measurement protocols were consistent with those of the ECC tensile performance test, as detailed in Section 2.2.3 of this paper.

### 3.2. Results and Analysis

#### 3.2.1. Experimental Phenomena

**Figure 5:**
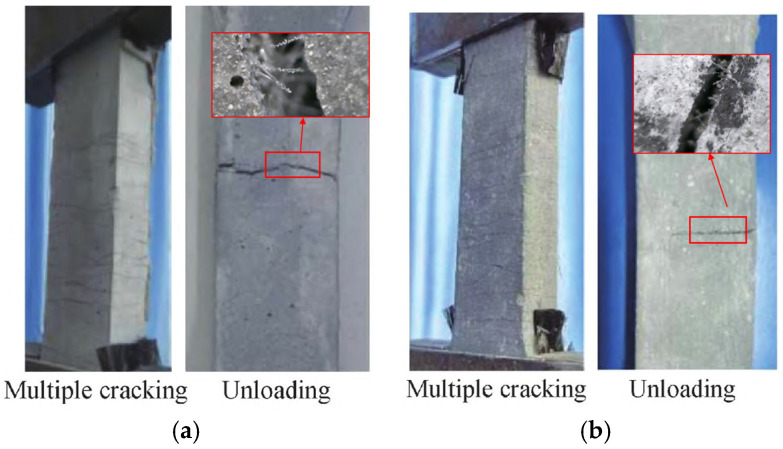
ECC and S-0.2-0.2 specimen cracking damage pattern: (**a**) ECC; (**b**) S-0.2-0.2.

All specimens underwent multiple-crack propagation during the loading process, and a single main crack formed in the failure stage for all specimens; after unloading, the microcracks around the main crack were effectively closed. During loading, microcracks propagated from both ends of the specimens toward the middle until reaching saturation. Upon reaching the ultimate stress, the specimens developed distinct main cracks and eventually lost their load-bearing capacity. Unlike ECC specimens, SMAF-ECC specimens exhibited superior main crack closure performance after unloading, with the crack width significantly reduced. This crack closure characteristic is consistent with the research results of Yang et al. [31] on SMAF-ECC under cyclic tension. Figure 5 illustrates the cracking failure modes of ECC and S-0.2-0.2 specimens. The results show that the main crack of the ECC specimen failed to close effectively after unloading, while the main crack of the S-0.2-0.2 specimen was effectively closed. This indicates that the knotted SMAF can achieve sufficient anchorage with the ECC matrix and exert superelasticity; after unloading, the restoring force generated by SMAF drives the reduction or complete closure of the main crack.

#### 3.2.2. Stress–Strain Curve

(1) SMAF-ECC specimens

**Figure 6:**
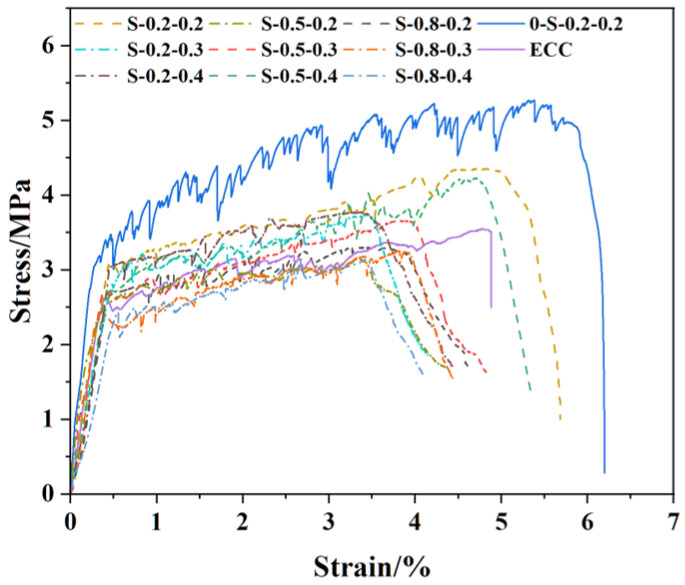
Stress–strain curves of ECC specimens and SMAF-ECC specimens.

As illustrated in Figure 6, the tensile stress–strain curves of all SMAF-ECC specimens exhibit a three-stage development pattern. In the initial loading stage, the stress and strain of SMAF-ECC specimens increase linearly until the initial cracking point is reached, where the tensile load is primarily sustained by the cement mortar matrix. During the middle loading stage, the matrix generates continuous microcracks, and PP/PVA fibers cooperate with SMAF to exert a bridging effect [30]. The stress fluctuates as the load increases until it reaches the ultimate tensile stress. This stage is characterized by distinct strain hardening behavior of the curve, with multiple fluctuations attributed to the continuous generation of microcracks within the matrix. In the strain softening stage, the curve starts to decrease after reaching the peak stress; at this point, PP and PVA fibers are fully depleted of their load-bearing capacity, and SMAF alone sustains the failure load until the specimen’s load-bearing capacity is lost.

(2) Comparative analysis of specimens

As can be seen from Figure 6, in contrast to the tensile stress–strain curves of conventional ECC specimens [11], the descending segment of the curve for SMAF-ECC specimens shows a slower decline rate, while that for ECC specimens is steep. This is because after the PP/PVA fibers completely cease to work, the two ends of the SMAF remain well anchored to the ECC matrix [37], which can continue to exert the bridging effect and effectively slow down the rate of stress attenuation. This indicates that the incorporation of SMAF is able to delay the damage development of the specimens [38]. The tensile stress–strain curves of specimen S-0.2-0.2 and specimen 0-S-0.2-0.2 (PP-free specimen) exhibit similar morphologies; however, the curve of the former presents less oscillation with a smaller amplitude than that of the latter. This is attributed to the relatively low content of PVA fibers in specimen S-0.2-0.2, which weakens its failure stress transfer capacity compared with specimen 0-S-0.2-0.2, thereby reducing the multiple-cracking characteristics.

#### 3.2.3. Tensile Performance Index

The tensile performance parameters of all tensile specimens derived from the tests are presented in Table 6. As shown in Table 6, the initial cracking stress of ECC specimens without SMAF is only 2.62 MPa, exhibiting low toughness. The incorporation of SMAF significantly enhances the initial cracking stress and ultimate tensile stress of certain SMAF-ECC specimens, with the initial cracking stress of most SMAF-ECC specimens increasing to the range of 2.66~3.06 MPa. Compared with SMAF-free ECC specimens, the initial cracking stress of each SMAF-ECC specimen with 0.2% SMAF volume content increases by 16.03~16.79%. This improvement trend is consistent with the research results of Gurbuz et al. [39]. Compared with the SMAF-free ECC specimen, only the ultimate tensile strain of specimen S-0.2-0.2 was improved by 2.87%, while that of specimen S-0.5-0.2 decreased by 31.35%. This is because the matrix designed for PVA/PP fibers is unlikely to achieve optimal matching with SMAF; thus, the incorporation of SMAF reduces the ductility of SMAF-ECC materials [40]. The ultimate tensile stress of all SMAF-ECC specimens with 0.8 mm diameter SMAF was lower than that of the SMAF-free ECC specimen, indicating that the negative effect of large-diameter SMAF outweighs its positive effect. Jafarypouria et al. found via finite element simulation that a larger fiber diameter increases the stress concentration factor in adjacent fibers, and fiber agglomeration further intensifies such stress concentration [41]. This finding also demonstrates that an increase in fiber diameter tends to induce a reduction in interfacial stress transfer efficiency. Specimen S-0.2-0.2 exhibited the optimal tensile performance, with its ultimate tensile stress and strain reduced by only 18.13% and 15.35% compared with specimen 0-S-0.2-0.2. This verifies that the SMAF-ECC with PP fibers partially replacing PVA fibers can maintain excellent tensile mechanical properties while reducing material costs.

#### 3.2.4. Analysis of Influencing Factors

(1) SMAF content

**Figure 7:**
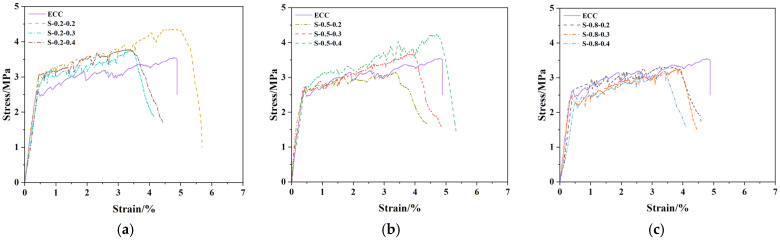
Stress–strain comparison curves of SMAF-ECC specimens with different SMAF contents: (**a**) SMAF diameter = 0.2 mm; (**b**) SMAF diameter = 0.5 mm; (**c**) SMAF diameter = 0.8 mm.

**Figure 8:**
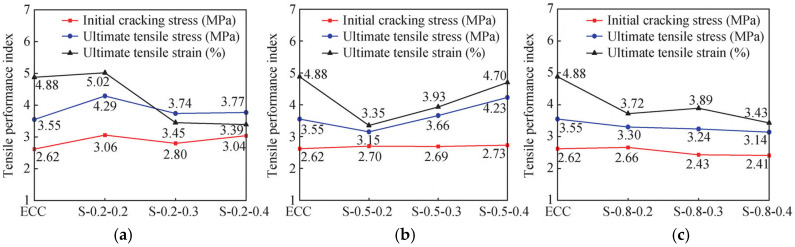
Comparison of tensile properties of SMAF-ECC specimens with different contents of SMAF: (**a**) SMAF diameter = 0.2 mm; (**b**) SMAF diameter = 0.5 mm; (**c**) SMAF diameter = 0.8 mm.

The stress–strain comparison curves and tensile performance indices of SMAF-ECC specimens with varying SMAF content are presented in Figure 7 and Figure 8. As observed in Figure 7, under the condition of a constant SMAF diameter, the stress–strain curves exhibit more frequent fluctuations as the SMAF content increases. This is attributed to the increased number of SMAFs, which enhances the internal fiber bridging capacity of the matrix [31] and results in more pronounced multi-cracking behavior. The influence of SMAF content on the initial cracking stress of SMAF-ECC specimens is not significant, with only a slight improvement observed for most specimens.

As shown in Figure 8a, the ultimate tensile stress of SMAF-ECC specimens with an SMAF diameter of 0.2 mm is significantly higher than that of SMAF-free ECC specimens. When the SMAF content is 0.2%, the specimens achieve the optimal ultimate tensile stress and strain, with the ultimate tensile strain reaching 5.02%. As the SMAF content increases further, the ultimate tensile strain of the specimens decreases. This is because when the SMAF diameter is small, excessive fiber content leads to difficulties in uniform fiber dispersion within the matrix, which impairs the compactness of the matrix and induces an increase in internal defects of the specimens.

It can be observed from Figure 8b that the ultimate tensile stress and strain of SMAF-ECC specimens with a 0.5 mm SMAF diameter increase with the rise in SMAF content. This phenomenon is consistent with the performance variation law of ultra-high-performance fiber-reinforced concrete (UHPFRC) reported by Paschalis et al. [42]. However, it is also found that the ultimate tensile strain of SMAF-ECC specimens decreases by 31.35%, 19.47%, and 3.69%, respectively, compared with the SMAF-free ECC specimen. A comparison with single-fiber-reinforced ECC specimens reveals that basalt fibers (BFs) can simultaneously improve the ultimate tensile stress and strain of specimens without strain reduction [43], while PVA fibers enable ECC to achieve a more significant improvement in tensile strength than BF [44].

As indicated in Figure 8c, for SMAF-ECC specimens with a 0.8 mm SMAF diameter, both the ultimate tensile stress and strain decrease with the increase in fiber content, and are even lower than the corresponding values of SMAF-free ECC specimen. In summary, the incorporation of an appropriate content of SMAF can improve the tensile properties of SMAF-ECC materials, whereas excessive SMAF addition results in the negative effect outweighing the positive effect, leading to a reduction in the ultimate tensile strain capacity of SMAF-ECC. Nevertheless, SMAF-ECC has a unique advantage: SMAF possesses both high ductility and deformation recovery performance. The crack closure coefficient of specimens with knotted SMAF is increased by 53% compared with that of conventional ECC, which is a distinctive property that PVA fibers, BFs, and other fibers do not have [27]. When the SMAF diameter is 0.2 mm or 0.8 mm, the SMAF-ECC specimens achieve optimal tensile performance at an SMAF content of 0.2 vol.%; whereas, for specimens with a moderate SMAF diameter of 0.5 mm, favorable tensile performance can be obtained only when the SMAF content is 0.4 vol.%.

(2) SMAF diameter

**Figure 9:**
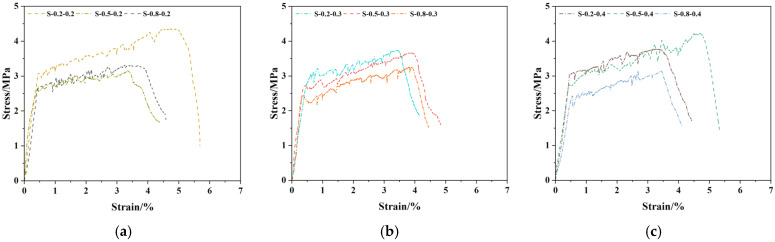
Comparative stress–strain curves of SMAF-ECC specimens with different diameters of SMAF: (**a**) φ(SMAF) = 0.2%; (**b**) φ(SMAF) = 0.3%; (**c**) φ(SMAF) = 0.4%.

**Figure 10:**
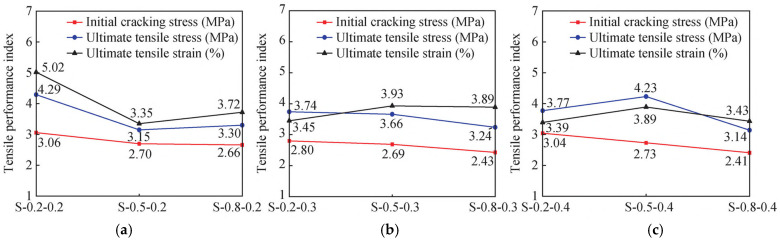
Comparison of tensile properties of SMAF-ECC specimens under different diameters of SMAF: (**a**) φ(SMAF) = 0.2%; (**b**) φ(SMAF) = 0.3%; (**c**) φ(SMAF) = 0.4%.

Figure 9 and Figure 10 present the stress–strain comparison curves and tensile performance indices of SMAF-ECC specimens with varying SMAF diameters. As observed in Figure 9, under the condition of constant SMAF content, the stress–strain curves exhibit more frequent fluctuations with the increase in SMAF diameter. This may be attributed to the reduced fiber count resulting from the increased SMAF diameter, which facilitates more uniform distribution of SMAF within the matrix. Consequently, the fiber bridging effect is better exerted, leading to more pronounced multi-cracking behavior [29].

As can be seen from Figure 10a, the ultimate tensile strain of specimens S-0.5-0.2 and S-0.8-0.2 decreased by 33.27% and 25.90%, respectively, compared with specimen S-0.2-0.2. This phenomenon may be explained by the fact that the deterioration effect induced by the increase in SMAF diameter is initially greater than the positive effect and then becomes smaller. On the one hand, at a constant fiber content, the number of SMAFs per unit volume decreases with the increase in fiber diameter. The reduction in fiber quantity weakens the fiber bridging effect; especially in the crack initiation stage, fewer fibers may fail to effectively inhibit the propagation of microcracks, thus leading to a decrease in initial cracking stress [45]. The research of Qiu et al. [46] also emphasized the influence of fatigue damage on the properties of fibers and fiber/matrix interfaces in ECC, further confirming the importance of the fiber bridging effect. On the other hand, the increase in fiber diameter can effectively improve the energy dissipation capacity of SMAF-ECC materials, thereby enhancing their tensile properties. Generally, thicker fibers can offer higher frictional resistance in the pull-out process, leading to more energy dissipation [47].

It can be observed from Figure 10b that at an SMAF content of 0.3 vol.%, the ultimate tensile stress of SMAF-ECC specimens shows a decreasing trend with the increase in SMAF diameter. The reduction in the tensile properties of the material may result from two reasons. First, as mentioned above, the decrease in fiber quantity weakens the fiber bridging effect. Second, the increase in fiber diameter may lead to a reduction in the bond strength between the SMAF surface and the ECC matrix. The decrease in bond strength impairs the pull-out efficiency of fibers from the matrix, which in turn affects the overall tensile properties and ductility of the material [48]. Yu et al. [49] also highlighted the importance of fiber–matrix interfacial performance for the tensile behavior of ECC. As shown in Figure 10c, at an SMAF content of 0.4 vol.%, the ultimate tensile stress and strain of SMAF-ECC specimens exhibit an increasing followed by a decreasing trend with the increase in SMAF diameter. This phenomenon may indicate that in the initial stage, the negative impact caused by the increase in SMAF diameter is less than the positive effect, while the negative impact becomes more significant with further increases in diameter. Such complex interactions are also reflected in the research on other fiber-reinforced composites. For example, in nanocomposites, the content of graphene nanoplatelets (GNPs) affects the mechanical, thermal, and viscoelastic properties of the composites, and their optimal content does not show a monotonically increasing or decreasing trend [50].

In summary, in the design of SMAF-ECC materials, it is necessary to optimize the fiber diameter according to the specific SMAF content to achieve the optimal tensile performance. Within the SMAF content range investigated in this study, the specimen with a fiber diameter of 0.2 mm achieves the best tensile performance at an SMAF content of 0.2 vol.%; when the SMAF content exceeds 0.2 vol.%, the specimen with a fiber diameter of 0.5 mm exhibits superior tensile performance.

## 4. Conclusions

In this study, a new type of SMAF-ECC with excellent mechanical properties and cost-effectiveness was prepared by partially replacing PVA fibers with PP fibers. In this study, through uniaxial tensile tests, the tensile mechanical properties of this SMAF-ECC were investigated, and the effects of SMAF volume content and diameter on these properties were compared and analyzed. The main conclusions are as follows:

(1) ECC was prepared by partially replacing PVA fibers with more economical PP fibers, and the resultant SMAF-ECC was fabricated by incorporating SMAF into the PP-modified ECC. This composite exhibits excellent tensile properties; specifically, the SMAF-ECC specimen with a 0.2 mm SMAF diameter and a 0.2 vol.% SMAF content attains the optimal tensile performance, with its ultimate tensile stress increased by 20.85% compared with SMAF-free ECC.
(2) SMAF content and diameter exert a significant effect on the tensile properties of SMAF-ECC specimens. The optimal fiber diameter is dependent on its volume content: a smaller fiber diameter (0.2 mm) is optimal at a low volume content (0.2 vol.%); whereas a moderate diameter (0.5 mm) is more suitable at high volume contents (0.3 vol.% or 0.4 vol.%).

## Figures and Tables

**Table 1:** Tensile properties of three different diameters of SMAF.

Test Item	National Standard Value	Experimental Value		
		0.2 mm Diameter	0.5 mm Diameter	0.8 mm Diameter
Elastic modulus [GPa]		29.2	28.7	25.4
Upper platform stress [MPa]	≥400	483	486	480
Tensile strength [MPa]	≥1000	1457	1433	1425
Elongation [%]	≥10	25	26	25
Residual deformation [%]	<0.5	0.17	0.18	0.18
Austenite phase transformation completion temperature [°C]		−20 ± 5	−20 ± 5	−20 ± 5

**Table 2:** Mechanical property indexes of PP and PVA fiber.

Fiber Type	Length [mm]	Diameter [mm]	Modulus of Elasticity [GPa]	Tensile Strength [MPa]	Density [g/cm^3^]
PP	12	0.03	3.5	500	0.91
PVA	12	0.04	39.0	1600	1.30

**Table 3:** Proportion of ECC.

Specimen No.	Mass					φ[%]*	
	Cement	Fly Ash	Quartz Sand	Water	Water Reducer	PP Fibers	PVA Fibers
ECC-0	0.5	2.0	0.1	0.11	0.008	/	2.0
ECC-1	0.5	2.0	0.1	0.11	0.008	2.0	/
ECC-2	0.5	2.0	0.1	0.11	0.008	1.0	1.0
ECC-3	0.5	2.0	0.1	0.11	0.008	0.8	1.2

φ[%]* represents the volume ratio of fiber to ECC material.

**Table 4:** Characteristic parameters of tensile stress–strain curves of ECC specimens with different mix ratios.

Specimen No.	Initial Cracking Stress [MPa]	Initial Cracking Strain [%]	Ultimate Tensile Stress [MPa]	Ultimate Tensile Strain [%]
ECC-0	2.63	0.44	4.50	5.12
ECC-1	1.83	0.97	2.51	2.14
ECC-2	3.03	1.60	3.95	2.39
ECC-3	2.62	0.39	3.55	4.88

**Table 5:** SMAF-ECC uniaxial tensile specimen table.

Specimen No.	SMAF Diameter [mm]	φ(SMAF) [%]	Number of Specimens
ECC	/	0	3
S-0.2-0.2	0.2	0.2	3
S-0.2-0.3	0.2	0.3	3
S-0.2-0.4	0.2	0.4	3
S-0.5-0.2	0.5	0.2	3
S-0.5-0.3	0.5	0.3	3
S-0.5-0.4	0.5	0.4	3
S-0.8-0.2	0.8	0.2	3
S-0.8-0.3	0.8	0.3	3
S-0.8-0.4	0.8	0.4	3
0-S-0.2-0.2	0.2	0.2	3

Note: Specimen number S-X-Y—S represents SMAF, X represents SMAF diameter, Y represents SMAF volume content; 0-S-0.2-0.2 specimens are based on ECC-0 without PP fibers.

**Table 6:** Characteristic parameters of stress–strain curves of SMAF-ECC tensile specimens.

Specimen No.	Initial Cracking Stress [MPa]	Initial Cracking Stain [%]	Ultimate Tensile Stress [MPa]	Ultimate Tensile Strain [%]
ECC	2.62	0.39	3.55	4.88
S-0.2-0.2	3.06	0.44	4.29	5.02
S-0.2-0.3	2.80	0.48	3.74	3.45
S-0.2-0.4	3.04	0.45	3.77	3.39
S-0.5-0.2	2.70	0.44	3.15	3.35
S-0.5-0.3	2.69	0.37	3.66	3.93
S-0.5-0.4	2.73	0.43	4.23	4.70
S-0.8-0.2	2.66	0.46	3.30	3.72
S-0.8-0.3	2.43	0.35	3.24	3.89
S-0.8-0.4	2.41	0.53	3.14	3.43
0-S-0.2-0.2	3.19	0.33	5.24	5.93

## Data Availability

The original contributions presented in this study are included in the article. Further inquiries can be directed to the corresponding author.
